# A Study of Fetal Outcomes and the Effect of Socioeconomic Status in Term Pregnancy With Oligohydramnios

**DOI:** 10.7759/cureus.91651

**Published:** 2025-09-05

**Authors:** Ravi Bhandari, Anjan Koirala

**Affiliations:** 1 Acute and General Medicine, Maidstone and Tunbridge Wells NHS Trust, Maidstone, GBR; 2 Radiodiagnosis, Lumbini Zonal Hospital, Butwal, NPL

**Keywords:** amniotic fluid index, fetal outcome, kuppuswamy classification, neonatal morbidity, nepal, oligohydramnios, socioeconomic status, term pregnancy

## Abstract

Background and objective

Oligohydramnios at term is associated with increased obstetric interventions and adverse perinatal outcomes. Socioeconomic status (SES) may influence these outcomes through disparities in healthcare access, health literacy, and antenatal care utilization. This study aimed to evaluate the association between SES and perinatal outcomes in women with term pregnancies complicated by isolated oligohydramnios.

Methods

This prospective observational study was conducted at Lumbini Provincial Hospital, Nepal, between April and October 2020. A total of 160 women with term oligohydramnios (amniotic fluid index [AFI] ≤5 cm, measured by a single radiologist using the four-quadrant technique) were enrolled. SES was classified using the Modified Kuppuswamy Scale. Because only one participant belonged to the Upper Middle class, 159 women in the Lower Middle and Upper Lower classes were included in comparative analyses. The primary outcome was a composite adverse neonatal outcome, defined as admission to the neonatal ICU (NICU), meconium aspiration, acute respiratory distress syndrome (ARDS), perinatal death, or Apgar score ≤7 at one minute. The secondary outcome was the mode of delivery. Statistical analyses included unadjusted comparisons and multivariable modeling using Firth’s penalized logistic regression for neonatal outcomes and logistic plus modified Poisson regression for cesarean delivery, adjusting for maternal age, parity, and comorbidity.

Results

Among 159 women analyzed, the mean maternal age was 25.6 ± 4.1 years, and 112 (70.4%) were primiparous. Socioeconomic distribution was as follows: 87 (54.7%) Lower Middle and 72 (45.3%) Upper Lower. Cesarean delivery occurred in 55 (34.6%) women, with no significant association with SES after adjustment (adjusted risk ratio [aRR] 0.97; 95% CI: 0.63-1.48). Composite adverse neonatal outcomes occurred in 9 (5.7%) cases, and SES was not significantly associated (adjusted odds ratio 0.79; 95% confidence interval [CI]: 0.19-3.35). Maternal comorbidity was independently associated with increased risk of adverse neonatal outcomes (adjusted odds ratio [aOR]: 10.87; 95% CI: 1.56-75.62).

Conclusions

In this cohort of 159 term pregnancies with oligohydramnios, no significant association was detected between SES and either neonatal outcomes or mode of delivery, with analyses restricted to the Lower Middle and Upper Lower classes. Maternal comorbidity, however, emerged as a significant predictor of adverse neonatal outcomes. These findings suggest that within a tertiary hospital setting, standardized monitoring and neonatal care may reduce the impact of socioeconomic disparities, while emphasizing the importance of optimizing maternal health to improve perinatal outcomes.

## Introduction

Oligohydramnios, defined as a reduced volume of amniotic fluid relative to gestational age, is most commonly diagnosed using ultrasonographic parameters such as the amniotic fluid index (AFI) or the single deepest vertical pocket (SDP) [1-4]. An AFI ≤5 cm or an SDP <2 cm is generally accepted as diagnostic [4]. Normal amniotic fluid volume is essential for fetal lung development, cushioning against cord compression, and overall intrauterine well-being [5]. The reported prevalence of oligohydramnios ranges from 0.5% to 5% depending on population and diagnostic criteria [6]. Although sometimes associated with maternal comorbidities or placental dysfunction, “isolated oligohydramnios” - defined as low amniotic fluid without other maternal or fetal pathology - remains a distinct clinical entity with implications for obstetric management [7].

Oligohydramnios at term has been associated with increased rates of labor induction, cesarean section, abnormal fetal heart rate patterns, meconium-stained amniotic fluid, low Apgar scores, and admission to the neonatal ICU (NICU) [8-10]. In severe cases, it has been linked to intrauterine growth restriction, stillbirth, and perinatal death [11]. However, evidence regarding its causal role remains inconsistent, with some authors suggesting oligohydramnios reflects underlying placental insufficiency while others argue it may be a benign variant [7-10]. In low- and middle-income countries (LMICs), resource constraints and limited access to intrapartum monitoring add complexity to the management of oligohydramnios [10]. Socioeconomic status (SES) has long been recognized as a determinant of maternal and perinatal health through its impact on healthcare access, nutrition, and health literacy [12]. Previous studies have shown SES to be associated with adverse pregnancy outcomes, including preterm birth, low birth weight, and perinatal mortality [13-15]. Yet, very little is known about how SES influences outcomes in pregnancies complicated specifically by isolated oligohydramnios at term, which represents a unique clinical scenario where the fetus is considered mature, but perinatal compromise may still occur.

The context of Nepal is particularly relevant as women who deliver at tertiary care hospitals, such as Lumbini Provincial Hospital, often differ from those delivering in rural or primary facilities in terms of demographic profile, referral patterns, and access to standardized neonatal services. Understanding how SES interacts with perinatal outcomes in this tertiary care setting is therefore important for assessing equity in maternal healthcare delivery. Despite global interest in oligohydramnios, most research has focused on preterm cohorts, where adverse outcomes are strongly influenced by immaturity itself. The term oligohydramnios, by contrast, is less extensively studied, and the interaction between SES and outcomes in this group has rarely been addressed. Moreover, maternal comorbidity, age, and parity may modify outcomes in pregnancies with oligohydramnios and require careful consideration in analysis. By focusing on term singleton pregnancies and using multivariable models to adjust for key confounders, this study sought to reduce bias and provide new evidence in a relatively underexplored population. The objective of this study was to assess whether maternal SES is associated with mode of delivery and adverse neonatal outcomes in term pregnancies complicated by isolated oligohydramnios, while accounting for maternal age, parity, and comorbidity.

## Materials and methods

Study design and setting

This was a prospective observational study conducted in the Department of Radiology at Lumbini Provincial Hospital, Butwal, Nepal, between April and October 2020. Ethical clearance and institutional approval were obtained from the Department of Radiology (approval letter dated Nepali calendar 077/01/01, equivalent to April 13, 2020). The study was authorized for the period April 17, 2020, to November 25, 2020. Verbal informed consent was obtained from all participants. The Institutional Ethics Committee specifically approved verbal consent due to COVID-19 restrictions and the limited literacy levels among some participants, ensuring that patients could understand the study information and provide informed agreement without unnecessary barriers.

Study population

**Figure 1:**
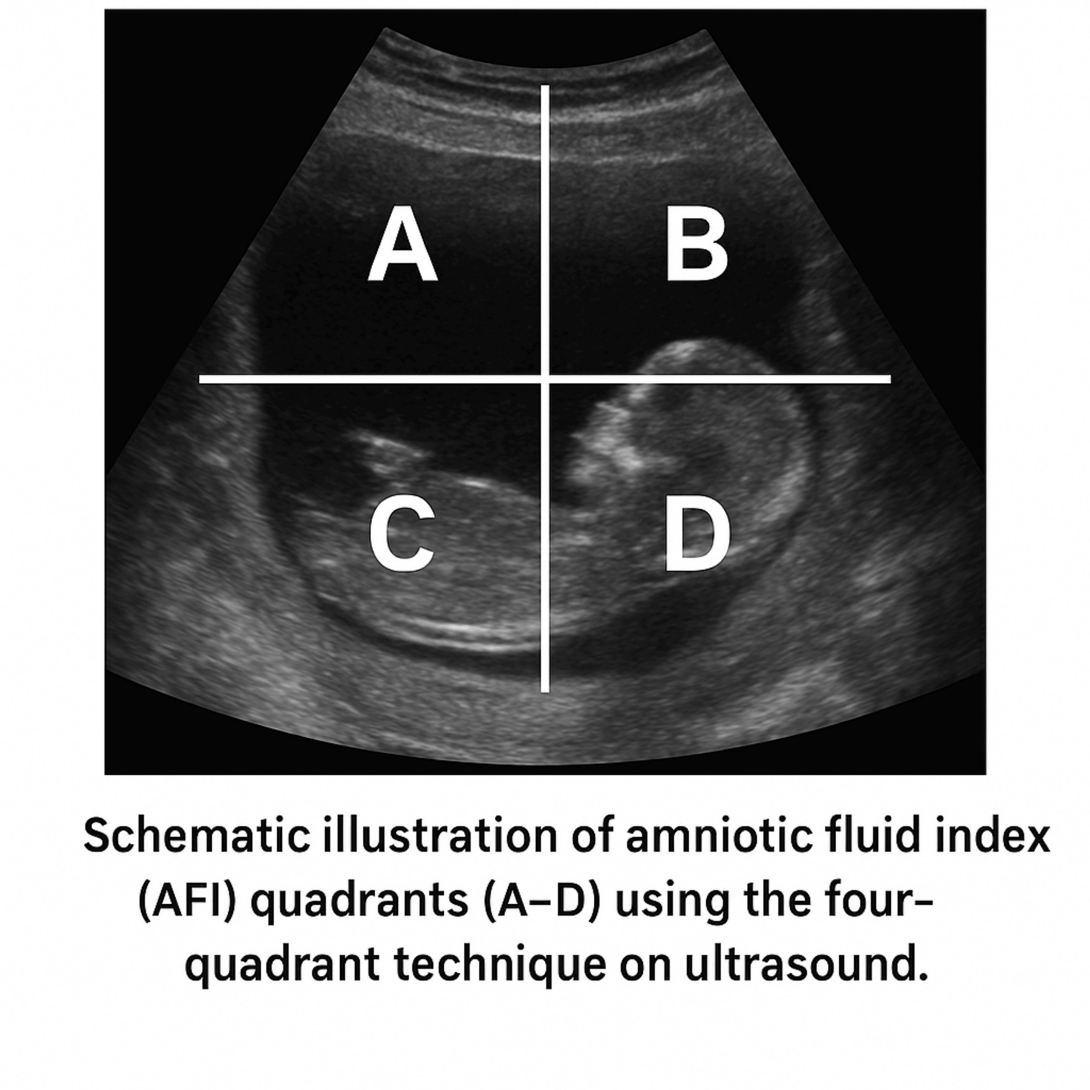
Schematic illustration of the AFI measurement using the four-quadrant technique The uterus is divided into four quadrants, and the deepest vertical pocket of fluid in each is measured (labeled A–D). The AFI is calculated as the sum of these four measurements AFI: amniotic fluid index

A total of 160 women with singleton term pregnancies were initially enrolled. Term pregnancy was defined as a gestational age between 37 and 42 completed weeks. AFI was assessed using the four-quadrant technique, in which the uterus is divided into four quadrants and the maximum vertical pocket of fluid in each is measured and summed as shown in Figure 1. Oligohydramnios was diagnosed on ultrasound when the AFI measured 5 cm or less using the four-quadrant technique [1,2]. All AFI measurements in this study were performed by a single radiologist to minimize inter-observer variability, with standardized protocols followed to ensure consistency.

The exclusion criteria were as follows: multiple gestation, gestational age <37 weeks, major congenital anomalies, polyhydramnios, and premature rupture of membranes. One participant classified as Upper Middle socioeconomic class was excluded from SES-based comparative analyses because of inadequate cell size. Thus, 159 women were included in final regression analyses (Lower Middle, n=87; Upper Lower, n=72). Baseline demographic and clinical characteristics are shown in Table 1.

**Table 1:** Baseline demographic and clinical characteristics of women with term oligohydramnios, stratified by SES SD: standard deviation; SES: socioeconomic status

Variable	Lower Middle (n=87)	Upper Lower (n=72)	Total (n=159)
Age, years, mean ± SD	25.41 ± 3.73	25.82 ± 4.46	25.60 ± 4.07
Primiparous, n (%)	63 (72.4%)	49 (68.1%)	112 (70.4%)
Multiparous, n (%)	23 (26.4%)	23 (31.9%)	46 (28.9%)
Chronic illness, n (%)	5 (5.7%)	1 (1.4%)	6 (3.8%)
Smoking, n (%)	0 (0.0%)	0 (0.0%)	0 (0.0%)

Socioeconomic status classification

SES was assessed using the Modified Kuppuswamy Scale (2020 update for Nepal) [16]. This scale combines education, occupation, and monthly family income (adjusted annually for inflation). Each domain is scored and summed, and the total determines the SES class. Women were initially classified into Upper Middle, Lower Middle, and Upper Lower classes; only the Lower Middle (Class III) and Upper Lower (Class IV) strata were analyzed.

Data collection

Data were prospectively recorded using a structured proforma. Maternal variables included age, parity (primiparous vs. multiparous), socioeconomic class, and chronic comorbidities such as hypertension, diabetes, or asthma. Smoking status was collected, but none reported smoking.

Ultrasound variables included AFI measurement and fetal presentation. Delivery details included mode of delivery (vaginal vs. lower segment cesarean section [LSCS]) and indication for cesarean. Neonatal outcomes included one-minute Apgar score, NICU admission (with indication and duration), meconium aspiration, acute respiratory distress syndrome (ARDS), and perinatal mortality. NICU admissions adhered to institutional neonatal protocols, which recommend admission for respiratory distress, suspected sepsis, meconium aspiration, or perinatal compromise, though the final decision rested with the neonatologist. Reasons for NICU admission are presented in Table 2, allowing readers to interpret heterogeneity within the composite neonatal outcome.

**Table 2:** Mode of delivery and frequency of composite adverse neonatal outcomes in women with term oligohydramnios, stratified by SES SES: socioeconomic status

Mode of delivery and composite adverse neonatal outcomes			
Vaginal delivery, n (%)	56 (64.4%)	47 (65.3%)	103 (64.8%)
Cesarean section, n (%)	30 (34.5%)	25 (34.7%)	55 (34.6%)
Fetal demise, n (%)	1 (1.1%)	0 (0.0%)	1 (0.6%)
Composite adverse neonatal outcome, n (%)	5 (5.7%)	4 (5.6%)	9 (5.7%)

Study variables

The primary exposure variable was maternal SES (Lower Middle vs. Upper Lower). The primary outcome was a composite adverse neonatal outcome, defined as the occurrence of at least one of the following: NICU admission, meconium aspiration, ARDS, perinatal death, or a one-minute Apgar score ≤7 [17]. The secondary outcome was mode of delivery, categorized as vaginal vs. cesarean delivery. Covariates considered as potential confounders included maternal age (continuous, in years), parity (primiparous vs. multiparous), and presence of chronic comorbidity (yes/no). Smoking was excluded due to a lack of variability in the sample.

Sample size calculation

The minimum required sample size was estimated using the single-proportion formula, n = (Z² × p × (1 - p)) / d², where Z = 1.96 for a 95% confidence level, p = 0.10 (estimated prevalence of oligohydramnios at term) [5], and d = 0.05 (absolute precision). Substitution gave: n = (1.96² × 0.10 × 0.90) / (0.05²) ≈ 138. To account for exclusions and improve statistical stability, 160 participants were recruited.

Statistical analysis

Data were analyzed using Python (v3.11; statsmodels package) and R (v4.5.1; logistf package). Continuous variables were summarized as mean ± standard deviation (SD) or median with interquartile range (IQR). Categorical variables were presented as absolute counts and percentages; 95% percent confidence intervals (CIs) for proportions were calculated using Wilson’s method [18].

The composite neonatal outcome was analyzed using Firth’s penalized logistic regression, which reduces small-sample bias and provides stable estimates when event counts are low. Adjusted odds ratios (aORs) with 95% CIs were reported [19]. The secondary outcome (cesarean delivery) was analyzed using both multivariable logistic regression (aORs) and modified Poisson regression with robust standard errors (aRRs), which directly estimate risk ratios for common outcomes. Both models adjusted for maternal age, parity, and comorbidity [20].

Model diagnostics included assessment of residuals and collinearity. Statistical significance was defined as p<0.05 (two-sided). Sensitivity analyses included restricting SES to two categories and repeating models with reduced covariates, confirming the robustness of findings. Results of the multivariable Firth's logistic regression for neonatal outcomes are shown in Table 3, and regression models for cesarean delivery are presented in Table 4.

**Table 3:** aORs with 95% CIs from Firth's logistic regression for predictors of composite adverse neonatal outcomes aOR: adjusted odds ratio; CI: confidence interval; SES: socioeconomic status

Predictor	aOR (95% CI)	P-value
SES (Lower Middle vs. Upper Lower)	0.79 (0.19–3.35)	0.75
Maternal age (per year)	1.00 (0.83–1.22)	0.98
Primiparous (vs. multiparous)	1.36 (0.24–7.78)	0.73
Comorbidity (yes vs. no)	10.87 (1.56–75.62)	0.016

**Table 4:** Multivariable logistic regression and modified Poisson regression models estimating aORs and aRRs for predictors of cesarean delivery aRR: adjusted risk ratio; aOR: adjusted odds ratio; CI: confidence interval; SES: socioeconomic status

Predictor	Logistic regression aOR (95% CI), p	Modified Poisson aRR (95% CI), p	
SES (Lower Middle vs. Upper Lower)	0.95 (0.49–1.84), p=0.88	0.97 (0.63–1.48), p=0.88	
Maternal age (per year)	1.03 (0.94–1.12), p=0.56	1.02 (0.96–1.07), p=0.55	
Primiparous (vs. multiparous)	0.79 (0.37–1.69), p=0.55	0.86 (0.54–1.39), p=0.55	
Comorbidity (yes vs. no)	1.00 (0.17–5.74), p=1.00	1.00 (0.33–3.08), p=1.00	

## Results

Baseline characteristics

A total of 159 women with term oligohydramnios were included in the final analysis, after exclusion of one participant from the Upper Middle socioeconomic class. The mean maternal age was 25.6 ± 4.1 years (range: 18-38 years). The cohort was predominantly primiparous (n=112, 70.4%), with multiparous women accounting for 46 (28.9%) cases. Numbers for parity categories sum to 158 rather than 159 due to the exclusion of the single participant from the Upper Middle socioeconomic class in comparative analyses. Chronic medical illness was present in six (3.8%) women, while none reported smoking. Socioeconomic classification by the Modified Kuppuswamy Scale revealed that 87 (54.7%) participants were in the Lower Middle class and 72 (45.3%) in the Upper Lower class, as shown in Table 1 [16].

Mode of delivery

Among all deliveries, 103 (64.8%) were vaginal and 55 (34.6%) were cesarean sections. The proportion of cesarean deliveries was nearly identical between the Lower Middle (n=30, 34.5%) and Upper Lower (n=25, 34.7%) groups, with no significant difference (p=0.88). Indications for cesarean section were varied, including non-reassuring fetal heart rate, meconium-stained liquor, failed induction, malpresentation, and cephalopelvic disproportion, without notable SES differences. Full distribution of delivery mode by SES is summarized in Table 2 [21,22].

Neonatal outcomes

The composite adverse neonatal outcome occurred in nine (5.7%) cases overall, with comparable rates in the Lower Middle (n=5, 5.7%) and Upper Lower (n=4, 5.6%) groups (p=0.97). Specific adverse events included five (3.1%) NICU admissions for respiratory distress, three (1.9%) cases of meconium aspiration, two (1.2%) admissions for suspected neonatal sepsis/fever, and one (0.6%) perinatal death. No infants had an isolated Apgar score ≤7 at one minute. Reasons for NICU admission are detailed in Table 2 [17,21]. The single fetal demise (0.6%) was included within the composite adverse neonatal outcome. 

Multivariable analysis of neonatal outcomes

Firth’s penalized logistic regression was used to account for the small number of adverse events. After adjusting for maternal age, parity, and comorbidity, SES was not significantly associated with composite neonatal outcomes (aOR: 0.79, 95% CI: 0.19-3.35, p=0.75). Maternal age and parity were also not significant predictors. The only significant predictor was the presence of maternal comorbidity, which was associated with a markedly increased risk of adverse neonatal outcome (aOR: 10.87, 95% CI: 1.56-75.62, p=0.016), as shown in Table 3 [19].

Multivariable analysis of mode of delivery

Both multivariable logistic regression and modified Poisson regression were used to assess predictors of cesarean delivery. SES was not significantly associated with cesarean delivery in either model (logistic regression aOR: 0.95, 95% CI: 0.49-1.84, p=0.88; Poisson regression aRR: 0.97, 95% CI: 0.63-1.48, p=0.88). Maternal age, parity, and comorbidity status were also not significant predictors of cesarean delivery, as detailed in Table 4 [20].

Summary of findings

In this cohort, adverse neonatal outcomes were infrequent, and cesarean rates were nearly identical across socioeconomic groups. After adjustment for maternal age, parity, and comorbidity, no significant association was detected between SES and either composite adverse neonatal outcomes or mode of delivery. Maternal comorbidity, however, was independently associated with an increased risk of adverse neonatal outcomes. These results suggest that within a standardized institutional care setting, socioeconomic disparities may not substantially influence immediate perinatal outcomes in pregnancies complicated by oligohydramnios [6-9,12,13,21].

## Discussion

This prospective observational study evaluated the association between maternal SES and perinatal outcomes in 159 women with term singleton pregnancies complicated by oligohydramnios. Using the Modified Kuppuswamy Scale, outcomes were compared between Lower Middle and Upper Lower socioeconomic groups, as the single participant from the Upper Middle class was excluded from comparative analyses. After adjustment for maternal age, parity, and comorbidity, no significant association was detected between SES and either composite adverse neonatal outcomes or cesarean delivery. In contrast, maternal comorbidity was independently associated with an increased risk of adverse neonatal events, underscoring the importance of maternal health status as a key determinant of neonatal outcomes in this population.

The absence of an SES effect contrasts with reports from other LMICs, where disadvantaged women frequently experience higher rates of operative delivery, neonatal morbidity, and mortality due to delays in health-seeking behavior and limited antenatal care [6,12,13]. Our findings suggest that when women deliver in a tertiary referral hospital with standardized intrapartum monitoring, evidence-based obstetric decision-making, and neonatal intensive care support, socioeconomic disparities in short-term perinatal outcomes may be minimized. The prognostic significance of isolated oligohydramnios is a matter of debate. Some studies report increased risks of meconium aspiration, respiratory distress, and perinatal death [6-9], whereas others suggest that oligohydramnios is more often a marker of placental insufficiency than a direct cause of morbidity [10,11]. In our cohort, adverse neonatal outcomes were infrequent (nine cases, 5.7%), with respiratory distress being the most common. Importantly, these events showed no socioeconomic gradient. The relatively low complication rate may reflect the protective effect of timely institutional delivery and immediate neonatal care, rather than the absence of risk associated with oligohydramnios itself.

Our deliberate focus on term pregnancies contributes novel evidence to an underexplored area. Much of the literature addresses preterm cohorts, where outcomes are dominated by complications of prematurity [14,15]. By restricting inclusion to 37-42 weeks, we minimized this source of heterogeneity and reduced confounding by prematurity. However, this choice may have masked broader socioeconomic effects on preterm birth and related morbidity. The method of diagnosing oligohydramnios also merits consideration. We used the amniotic fluid index (AFI ≤5 cm), which remains widely practiced [1,2], but evidence suggests AFI may overdiagnose low fluid and prompt unnecessary interventions, whereas the single deepest pocket (SDP <2 cm) is more specific [21]. Reliance on AFI alone is therefore a limitation, and future studies should validate findings using both AFI and SDP.

Defining adverse neonatal outcome as a composite endpoint provided clinical breadth but introduced heterogeneity. To address this, we reported the individual components separately: NICU admission for respiratory distress (3.1%), meconium aspiration (1.9%), neonatal sepsis or fever (1.2%), and perinatal death (0.6%). No infants had an isolated low one-minute Apgar score [17]. This breakdown allows readers to interpret the relative contribution of specific complications within the composite outcome. Our study also highlights the impact of maternal comorbidity, which emerged as a significant predictor of neonatal complications (aOR: 10.87). The wide CI around this estimate reflects the small number of adverse events, a common limitation in sparse-data analyses despite the use of penalized regression. This finding emphasizes the importance of screening and optimizing chronic conditions such as hypertension and diabetes, which may exert greater influence on perinatal outcomes than socioeconomic background in settings where standardized intrapartum care is available [19].

This study has a few limitations. This was a single-center study, which limits the generalizability of its findings. Women delivering in a tertiary hospital may differ from those in rural or primary facilities, where SES-related barriers could have a greater impact. Second, the underrepresentation of higher socioeconomic classes restricted analysis across the full SES spectrum. Third, although we adjusted for age, parity, and comorbidity, residual confounding from factors such as nutrition, antenatal care, and other social determinants cannot be excluded [12]. Fourth, the use of only the one-minute Apgar score may underestimate neonatal compromise, as the five-minute score is more predictive of longer-term outcomes [22]. Fifth, reliance on AFI rather than SDP may have led to overdiagnosis of oligohydramnios [21]. Finally, although powered for moderate associations, the modest sample size may have limited our ability to detect subtle effects.

Despite these limitations, the study has notable strengths. The prospective design minimized recall bias, and the use of a validated SES scale ensured systematic socioeconomic classification [16]. All AFI measurements were performed by a single radiologist with a standardized protocol, minimizing inter-observer variability. Application of Firth’s logistic regression for sparse events strengthened validity [19], and the use of both logistic and Poisson regression models confirmed the consistency of findings for cesarean delivery [20]. In summary, our results suggest that within a tertiary referral hospital, socioeconomic disparities may not substantially influence immediate perinatal outcomes in pregnancies complicated by oligohydramnios. Instead, maternal comorbidity appears to play a more critical role. Larger, multicenter studies that include both term and preterm pregnancies and apply both AFI and SDP diagnostic criteria are warranted to validate these findings and explore their relevance across diverse healthcare settings.

## Conclusions

In our cohort, we found no significant association between maternal SES and either composite adverse neonatal outcomes or mode of delivery after adjustment for age, parity, and comorbidity. In contrast, maternal comorbidity emerged as an independent risk factor for adverse neonatal outcomes, underscoring the importance of optimizing maternal health in this setting. By focusing exclusively on term pregnancies, this study contributes new evidence to an area that has received relatively little attention compared with preterm cohorts. The findings suggest that within a tertiary referral hospital equipped with standardized intrapartum monitoring and neonatal services, socioeconomic disparities may exert less influence on short-term perinatal outcomes. Nevertheless, the underrepresentation of higher socioeconomic groups, reliance on the amniotic fluid index alone, and the single-center design limit the generalizability of our findings. Larger, multicenter studies incorporating both AFI and SDP diagnostic criteria, and including preterm as well as term pregnancies, are needed to validate these findings and clarify the broader role of socioeconomic factors in perinatal health.
